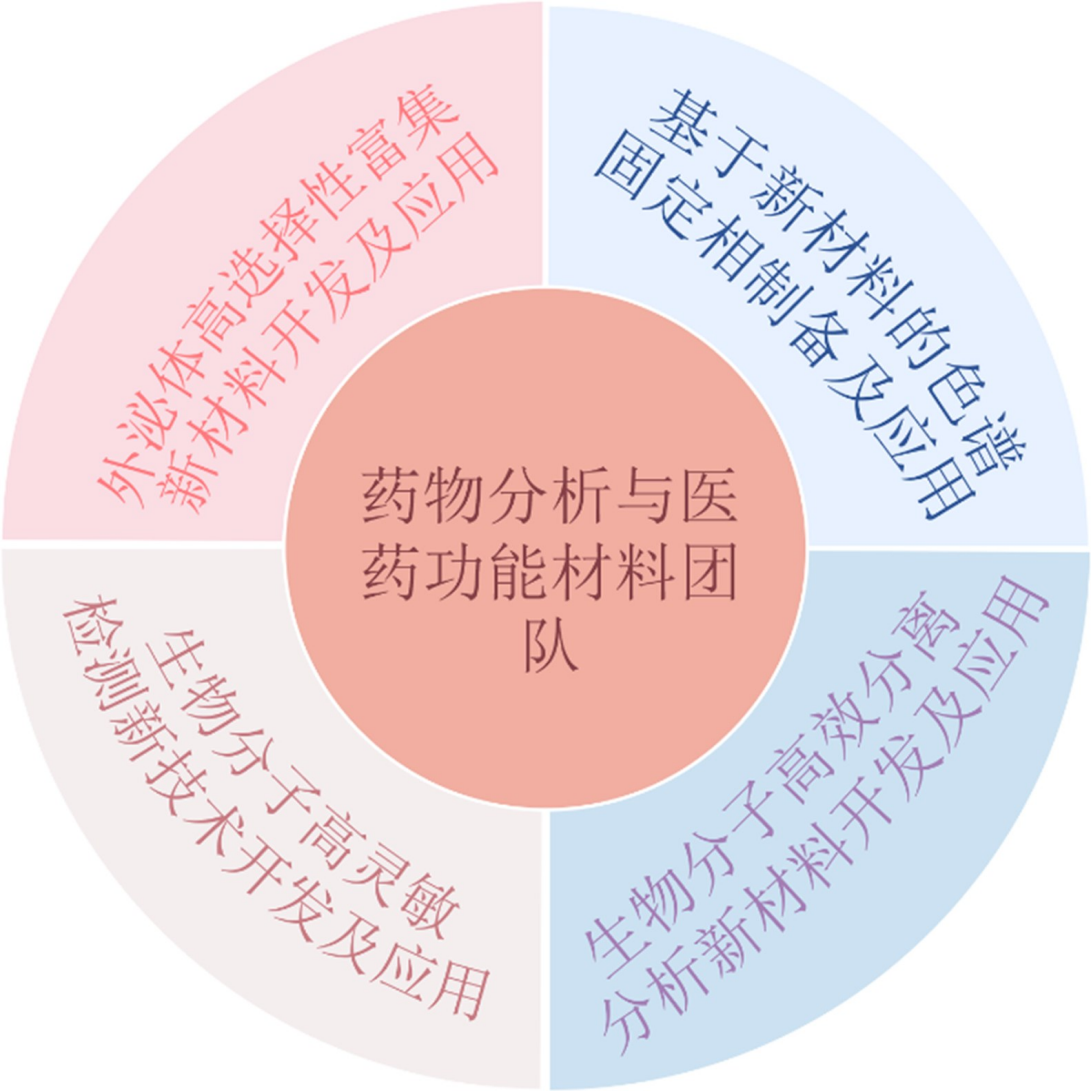# 基于亲和作用力的外泌体高效分离方法的经典策略和研究进展

**DOI:** 10.3724/SP.J.1123.2024.11004

**Published:** 2025-05-08

**Authors:** Haiyan WANG, Peijuan XIE, Xiaoqiang QIAO, Liyuan ZHANG

**Affiliations:** 1.河北大学药学院, 河北 保定 071002; 1. School of Pharmacy, Hebei University, Baoding 071002, China; 2.深圳综合粒子设施研究院, 广东 深圳 518107; 2. Institute of Advanced Science Facilities, Shenzhen 518107, China

**Keywords:** 外泌体, 生物标志物, 亲和作用, 分离富集, 研究进展, exosomes, biomarkers, affinity interaction, isolation and enrichment, research progress

## Abstract

外泌体是大多数细胞分泌的细胞外囊泡的一个特殊亚类,存在于血液、尿液、唾液、羊水、乳汁等各种生物体液中,也存在于各种组织和细胞间隙中,是细胞间通讯的重要介质。越来越多的研究表明,基于外泌体的液体活检有望成为疾病早期诊断、治疗监测以及预后评估的重要手段,高质量外泌体的分离则是保障后续相关诊断及治疗结果准确性和可重复性的前提和保障。然而,由于外泌体在样本中丰度较低,且与体液中的类似成分(如细胞碎片、蛋白质)共存,如何在复杂的生物样本中实现其高效的分离仍具有挑战性。目前,基于外泌体的物理、化学和生物特性,已发展出多种外泌体分离技术。近年来,基于亲和作用力的外泌体高效分离方法克服了传统分离方法的局限性和弊端,在科研和临床中得到了广泛应用。本文聚焦外泌体分离富集领域,系统综述了近年来基于亲和作用力的外泌体分离富集方法,并对外泌体分离富集方法的发展前景进行了分析和展望,期望为外泌体分离方法新策略的构建及应用提供参考。

## Abstract (en)

Exosomes form a subclass of extracellular vesicle that are secreted by most cells and found in nearly all body fluids, including blood, urine, saliva, amniotic fluid, and milk, as well as in various tissues and intercellular spaces. Exosomes have recently been recognized as crucial intercellular communication mediators, and an increasing number of studies have shown that exosomes are important liquid-biopsy tools that play irreplaceable roles in the diagnosis, prognosis, and treatment of diseases. The ability to isolate high-quality exosomes is a prerequisite for diagnosing and subsequently treating diseases in an accurate and repeatable manner. However, efficiently isolating exosomes from complex biological samples is challenging owing to their relatively low abundances and interference from non-vesicular macromolecules (such as cell debris and proteins). To date, various isolation techniques based on the physical, chemical, and biological characteristics of exosomes have been developed. Indeed, efficient affinity-interaction-based methods have recently overcome the limitations and drawbacks of traditional exosome isolation methods and are widely used in scientific research and clinical applications. This review focuses on exosome isolation and enrichment, and systematically reviews recent research progress on efficient isolation methods based on affinity interactions. Developmental prospects of exosome isolation and enrichment directions are analyzed with the aim of providing a reference for the construction and use of new exosome-isolation strategies.

外泌体是一种几乎所有活细胞都分泌的纳米级细胞外囊泡,存在于几乎所有生物体液中,是细胞间通讯的重要介质^[1]^。外泌体组成成分多样,包括核酸、蛋白质、脂质、氨基酸和代谢物,可动态反映其细胞来源的变化,能够将亲代细胞的特异性成分转移到受体细胞中^[2⇓-4]^。如图1所示,外泌体与免疫应答、病毒致病、心血管疾病、中枢神经系统相关疾病以及癌症进展等息息相关^[5⇓-7]^。由于外泌体包含DNA、RNA和蛋白质,基于外泌体的无创液体活检技术已逐渐成为肿瘤临床诊断的新前沿^[8]^。

**图1:**
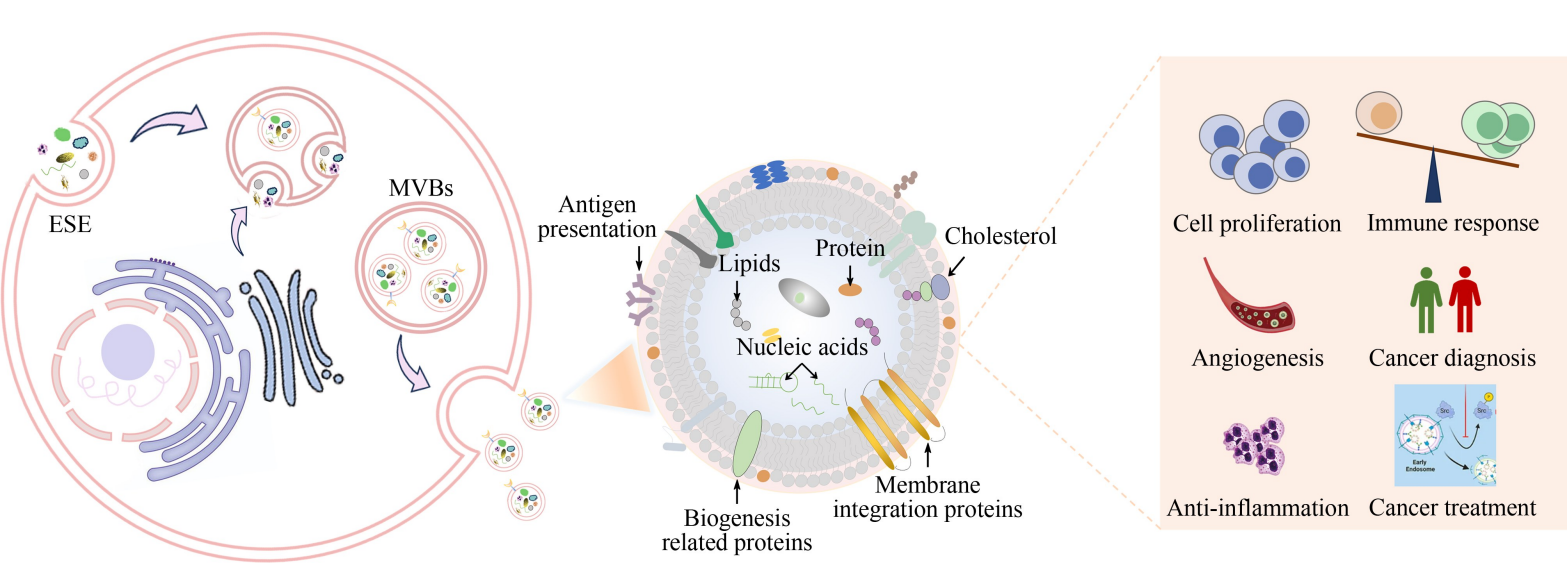
外泌体的生物功能

目前,外泌体的临床应用潜力开发主要受限于以下几点:(1)外泌体具有异质性,不同来源的外泌体,其膜的组成以及其内容物具有显著差异;(2)外泌体粒径小(纳米级)、密度低(1.10~1.19 g/mL)且带负电荷,其粒径和密度与体液中共存的其他成分(如细胞碎片、可溶性蛋白和脂蛋白质等)类似,常规的方法难以实现有效分离;(3)外泌体的分离时间和分离温度会显著影响外泌体的稳定性、内容物成分以及其粒径分布。随着外泌体分离鉴定方法的不断发展,许多分离方法被成功应用于外泌体的分离富集。

本文对基于亲和作用力的外泌体分离策略研究进展进行了综述,分别介绍了基于免疫、脂质膜、亲疏水以及微流控技术的亲和富集策略,最后探讨了亲和作用与常规外泌体分离技术结合应用于外泌体分离的潜力。

## 1 基于亲和作用力的外泌体分离富集方法

### 1.1 基于免疫亲和的富集法

免疫亲和富集法是一种基于外泌体表面特异性标志物的分离纯化技术^[9⇓-11]^。由于其具有高特异性、高纯度和易于纳入下游分析等优点,免疫亲和富集策略被广泛用于外泌体的分离捕获^[12]^。外泌体脂质膜表面富含蛋白质和受体,包括外泌体常规标志物和特异性标志物。其中,在亲和富集中最常用的外泌体标志物为膜蛋白,如CD9、CD63、CD56、CD81、CD82、CD91、CD105、CD147及CD151等^[13⇓-15]^。此外,通过靶向肿瘤相关的外泌体蛋白,如EpCAM和AntiA33,可以实现肿瘤来源外泌体的高效分离^[16]^。

近年来,基于与外泌体表面磷脂酰丝氨酸(PS)的特异性结合,研究人员开发了一系列针对外泌体的免疫亲和富集新策略^[17⇓⇓-20]^。如跨膜T细胞免疫球蛋白黏蛋白(Tim4),因其对PS的高亲和力,被发展作为亲和配体,用于外泌体的高效分离。本课题组发展了基于II-01MOFs基质的Tim4@ILI-01免疫亲和材料和磁性核壳Fe_3_O_4_@SiO_2_-ILI-0l@Tim4材料。其中Tim4@ILI-01免疫亲和材料对外泌体的捕获效率高达85.2%,是超速离心法(UC)的5.2倍。基于Fe_3_O_4_@SiO_2_-ILI-0l@Tim4材料建立的纳米液体活检技术,则实现了高通量、无创外泌体的分离富集,并成功应用于肺癌的早期诊断,且具有良好的特异性和超高的灵敏度(图2)^[21,22]^。Yang等^[19]^基于固定化肽配体与外泌体表面PS之间的特异性相互作用,提出了一种新型的外泌体分离策略,可以在短时间内高效回收完整的外泌体,富集的血清外泌体纯度远高于超滤法。Lu等^[23]^发展了基于PS的印迹聚合物纳米颗粒,并探讨了其应用于5种人体体液(血浆、尿液、羊水、脑脊液和唾液)中外泌体快速高效分离和代谢组学研究的可行性。结果发现,基于外泌体的代谢产物分析可以实现“追本溯源”。进一步对肝细胞癌患者临床血浆样本的外泌体代谢物进行分析,发现血浆外泌体中多种代谢物的表达水平与肝细胞癌的发生发展密切相关。

**图2:**
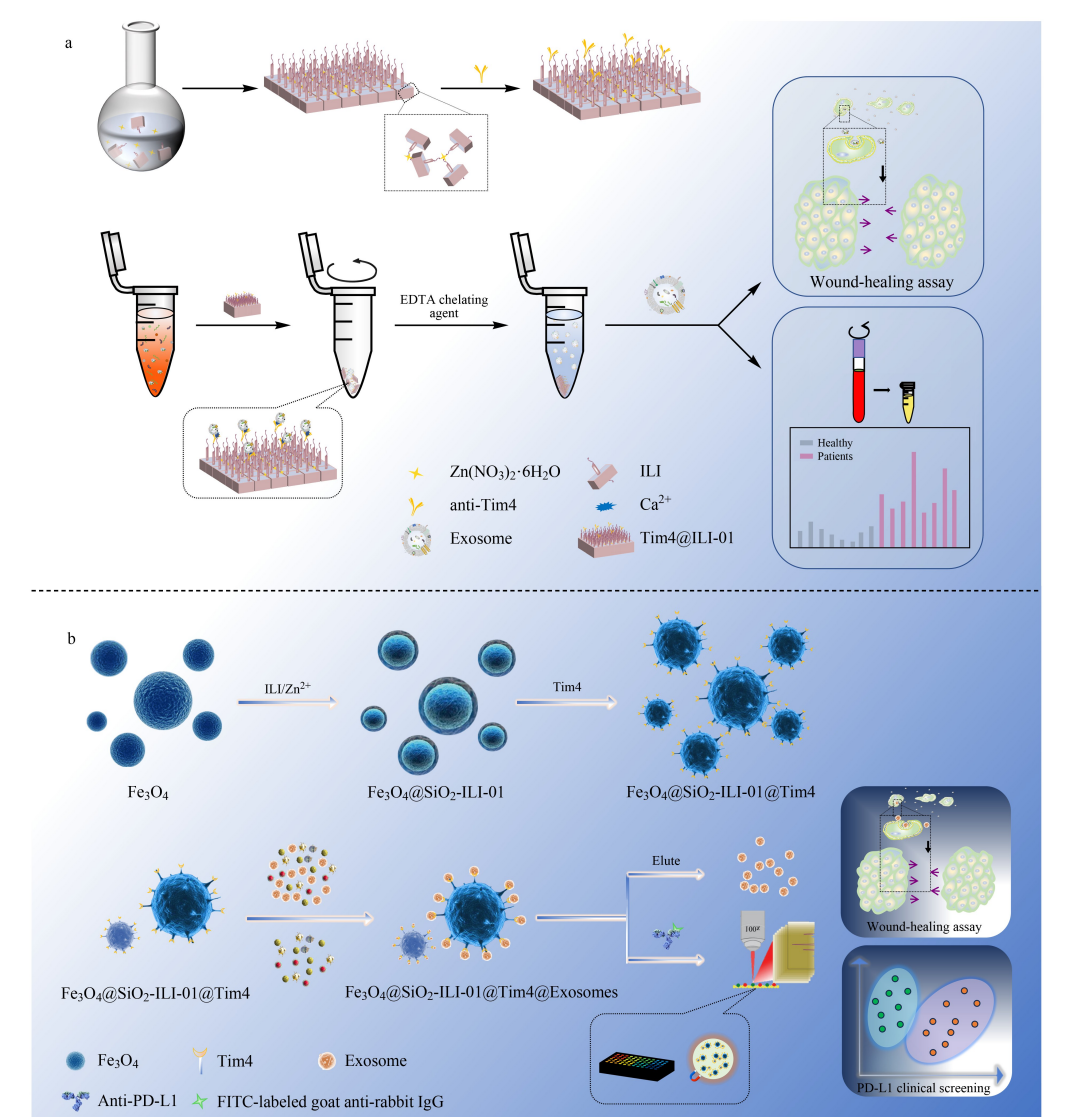
(a)Tim4@ILI-01和(b)Fe_3_O_4_@SiO_2_-ILI-0l@Tim4材料用于外泌体富集及下游分析的示意图^[21,22]^

基于免疫亲和作用力的外泌体分离技术,常常需要将特异性外泌体抗体或特异性外泌体结合分子固定于基质载体表面,如微球、金属有机框架(MOFs)、智能响应聚合物或微流控芯片等。抗体包被的基质载体可特异性结合相应的外泌体,进一步通过磁性分离、低速离心等方法可实现外泌体的分离和纯化。但基于固体基质的非均相性分离方法常存在界面传质阻力和高空间位阻,从而降低捕获效率。当处理极低丰度的外泌体时,该方法存在回收率低、重现性差、非特异性吸附率高等困扰。为解决该问题,本课题组^[24]^开发了一种基于水溶性pH响应聚合物和主客体相互作用的外泌体分离纳米系统(pH-HGN),以实现生理pH条件下外泌体的均相分离(图3)。该pH-HGN可在生理pH条件下的水溶液中,利用pH-HGN表面键合的抗CD63抗体实现外泌体的亲和均相捕获。随后,通过提高富集体系pH值,即可诱导pH-HGN-外泌体偶联物的快速自组装,进而实现简单高效的外泌体分离。最后,利用*α*-环糊精(*α*-CD)即可实现外泌体的均相高效洗脱。pH-HGN法有效结合了基于聚合物均相分离的高效性和基于免疫亲和法的高特异性。所建立的pH-HGN进一步成功应用于不同病理类型和分期的肺癌患者的高效区分,为基于外泌体的快速、无创液体活检开创了一条新途径。

**图3:**
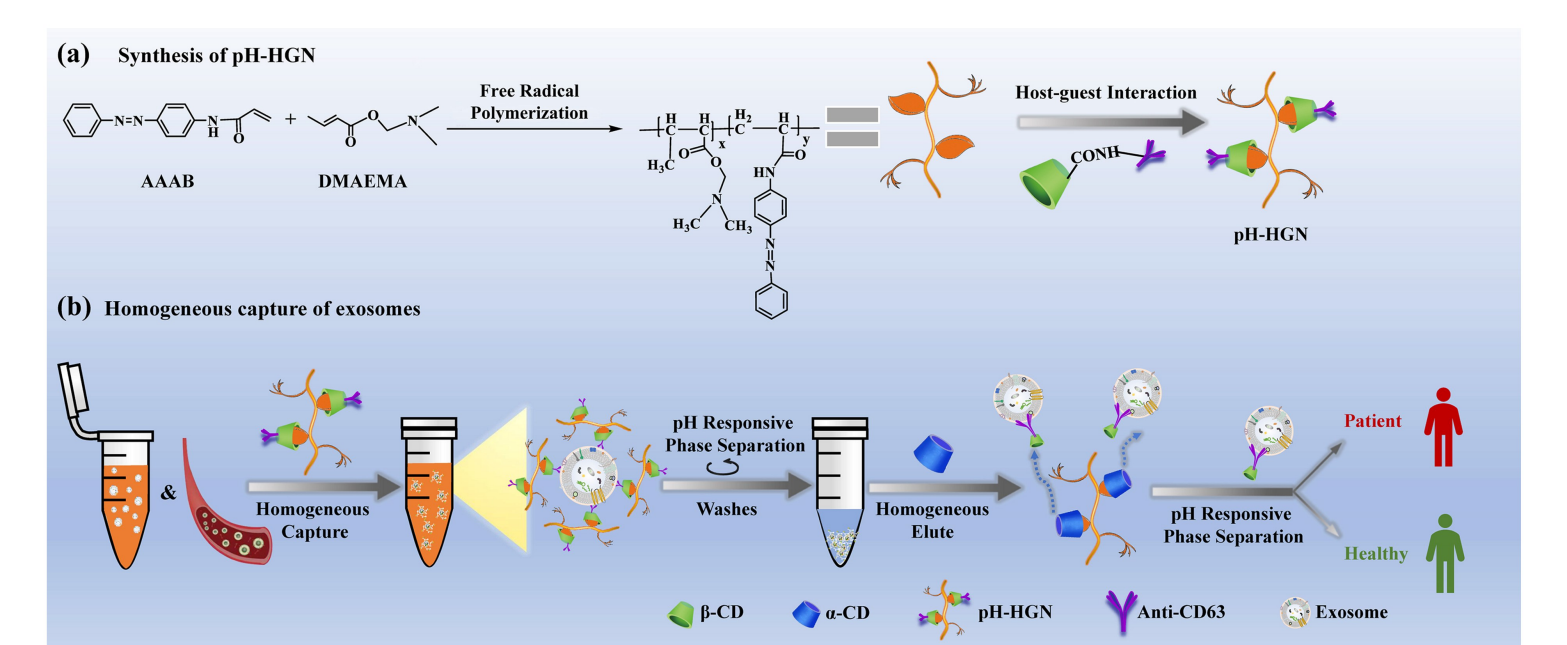
外泌体分离纳米系统富集外泌体^[24]^

### 1.2 基于脂质膜结构的亲和富集方法

外泌体是由细胞内陷形成的具有脂质双分子层结构的小囊泡,表面富含带负电荷的磷脂。在外泌体膜结构中,两亲性磷脂构成外泌体脂质双分子层的主要成分,位于膜外表面的亲水磷酸盐头可以与一些金属氧化物(如TiO_2_、ZrO_2_)特异性结合。基于此,Kanao等^[25]^将TiO_2_粒子与海绵状聚合物杂交,开发了一种新型外泌体纯化平台(TiO_2_-BSA-SPM)。TiO_2_-BSA-SPM可在1 h内完成各种生物样品中外泌体的分离(图4a)。其外泌体回收率是UC法的130.7倍,有望成为通用型外泌体富集平台。Zhang等^[26]^设计了结合CD63适配子的磁性TiO_2_纳米颗粒,在10 min内成功分离并捕获了92.6%的尿外泌体。Li等^[27]^基于外泌体膜上的磷脂酰胆碱(PC)和磁珠上修饰的磷酸胆碱(CP)之间的可逆两性离子配位作用,提出了一种可逆的两性离子协同策略,设计制备了CP修饰的磁珠(MB@CPs),用于快速(<30 min)从各种生物体液中提取外泌体。经对比,基于MB@CPs策略富集得到的外泌体的产量和纯度均优于传统的UC法(图4b)。稀土元素Eu中*f*轨道的阶数较高,对外泌体磷脂双层膜表面的磷酸基具有较强的吸附作用。Wu等^[28]^合成了核壳磁性纳米材料Fe_3_O_4_@SiO_2_@Eu_2_O_3_,实现了血浆中外泌体的快速分离。该磁性纳米材料较超速离心法,具有富集时间更短和操作更方便等优点(图4c)。

**图4:**
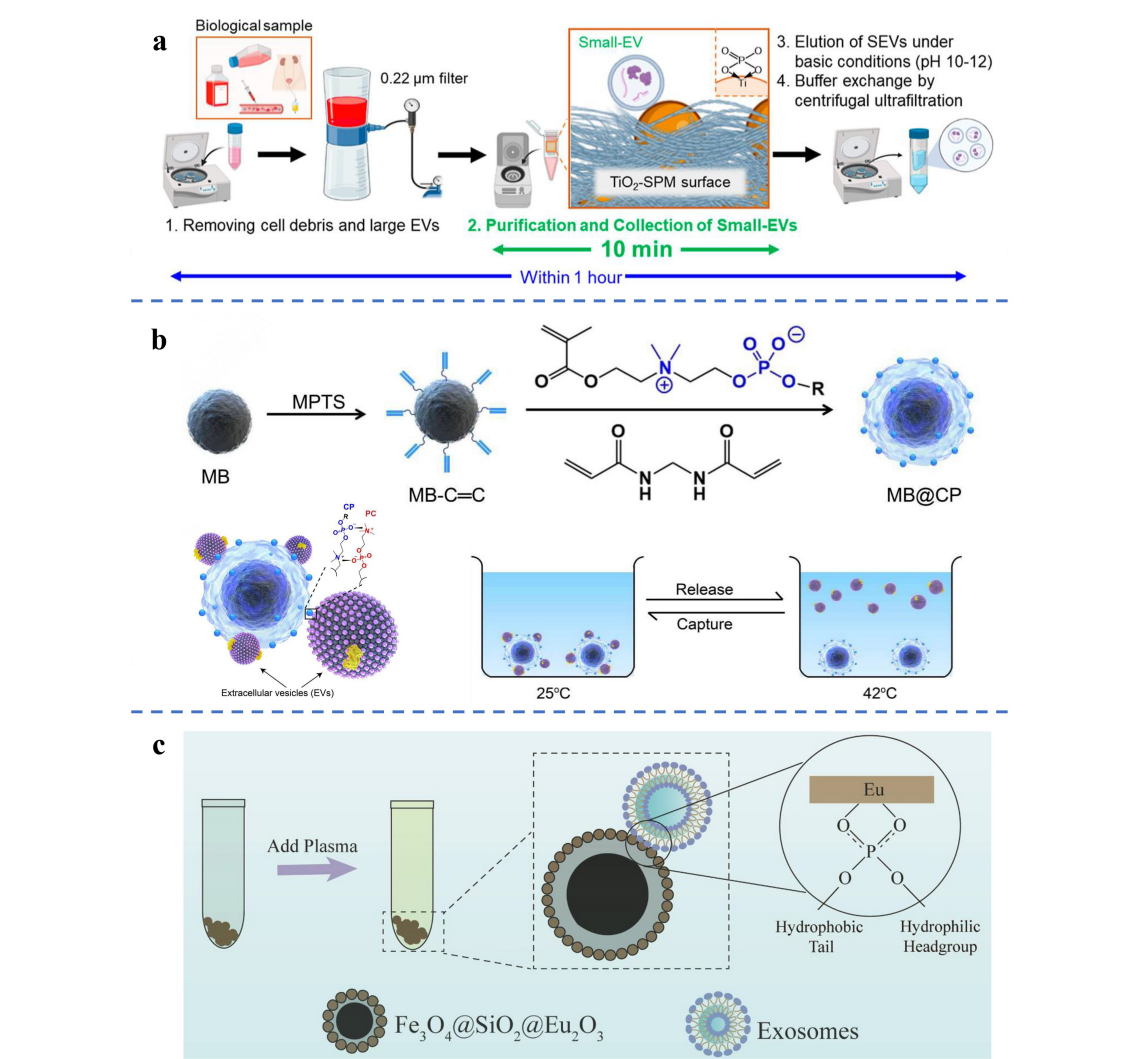
(a)基于TiO_2_-BSA-SPM的外泌体分离机制^[25]^; (b)外泌体通过高亲和力的CP-PC相互作用黏附在MB@CP上^[27]^; (c)Fe_3_O_4_@SiO_2_@Eu_2_O_3_纳米材料富集血浆中的外泌体^[28]^

基于外泌体膜结构的亲和富集方法几乎不依赖于外泌体表面蛋白,可以避免外泌体本身的异质性造成的低效率和低产量。但是,由于该方法主要基于脂质双层结构,分离的外泌体容易受到其他具有膜结构的生物体杂质的污染。

### 1.3 基于亲疏水相互作用的外泌体亲和分离方法

传统外泌体分离方法虽然应用最为广泛,但也存在样本消耗大、外泌体损伤、纯度低、耗时长等缺点,难以满足当前日益增长的研发需求。近年来,一些新兴的分离技术被提出并得到了迅速的发展,具有高纯度、高通量和低样品量的特点。本课题组开发了基于单宁酸(TA)自组装三维网络的SiO_2_@BSA@Fe-TA_6_材料。SiO_2_@BSA@Fe-TA_6_材料基于TA对外泌体表面糖萼的无标记亲水相互作用,实现了外泌体的通用型无标记捕获。进一步结合近红外光谱分析技术(NIRS)和化学计量学,SiO_2_@BSA@Fe-TA_6_成功实现了基于外泌体的肺癌患者液体活检临床诊断和病理分型,其对非小细胞肺癌和小细胞肺癌分型的准确率可达87.1%^[29]^。此外,SiO_2_@BSA@Fe-TA_6_的制备方案简单高效,单次制备的材料量即可满足同时处理多达500个血浆样本的使用需求,为大规模产业化的外泌体捕获提供了新方法(图5a)。基于不同类型的亲和作用,本课题组目前已发展了4种外泌体富集方法,详细对比见表1。

**图5:**
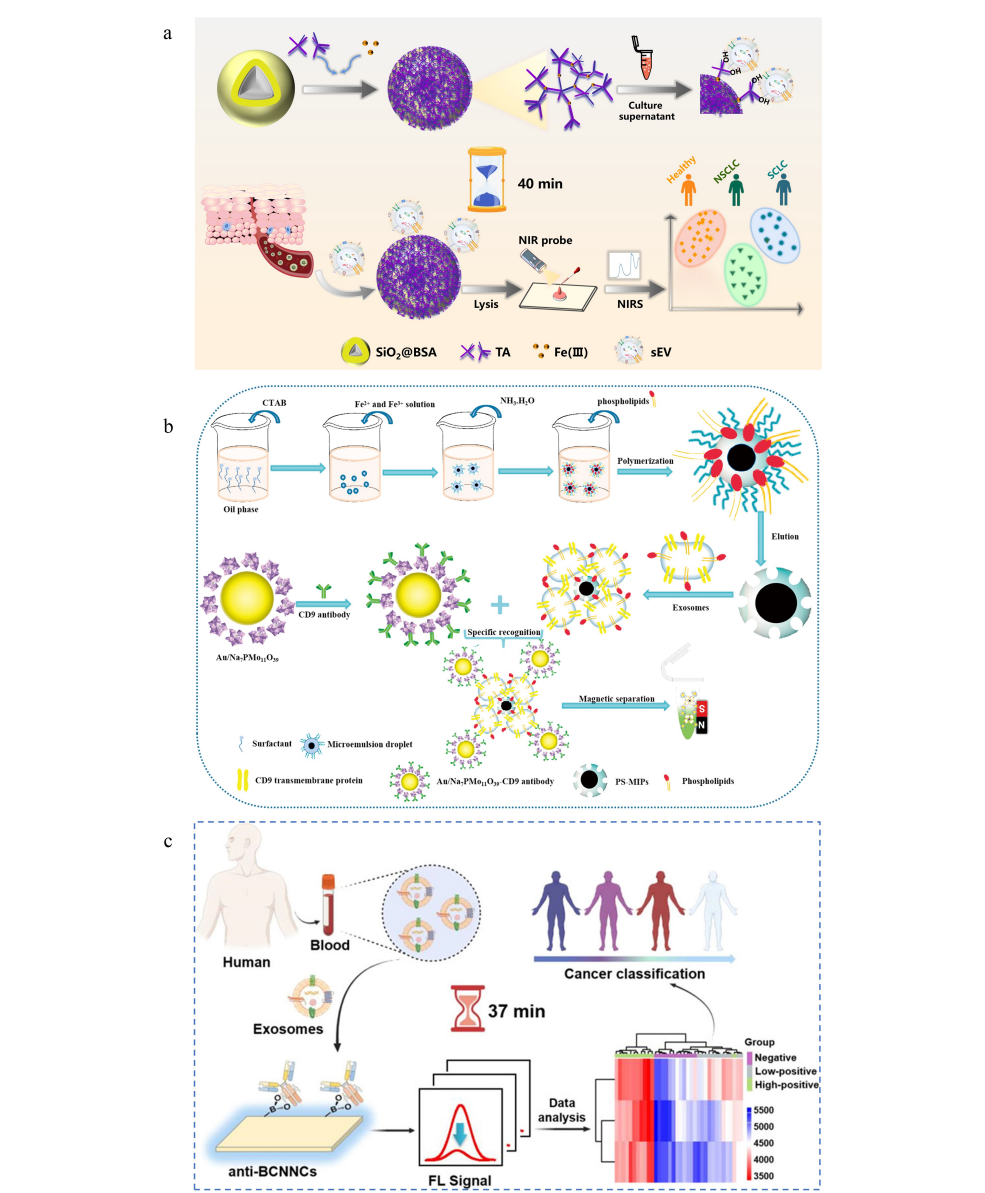
(a)SiO_2_@BSA@Fe-TA_6_在外泌体分离及下游分析中的应用^[29]^; (b)基于PS-MIPs-NELISA-SERS的外泌体检测平台^[31]^; (c)基于g-C_3_N_4_纳米片(BCNNS)的外泌体分离检测平台^[32]^

**表1:** 本课题组发展的基于亲和作用的外泌体富集方法比较

Approach	Capture efficiency/%	Advantages	Insufficiencies	Applications	Ref.
Tim4@ILI-01	85.2	high capture efficiency; easy nondestructive release; good biological activities of the captured exosomes	potentially exosome loss due to multiple mechanical centrifugation steps	sera of healthy persons and lung adenocarcinoma patients	[22]
Fe_3_O_4_@SiO_2_- ILI-0l@Tim4	90.3	high-efficiency and high-throughput exosome capture and release; simple and quick sample handling	increased economic cost due to the employment of Tim4	high-throughput exosomal PD-L1 screening for accurately predicting anti-PD-1 response	[21]
pH-HGN	90.2	highly efficient homogeneous capture and rapid recovery of intact exosomes	biological interference of enriched exosome in clinical treatment due to the conjugation of anti-CD63 antibody	differentiating lung-cancer patients from healthy persons	[24]
SiO_2_@BSA@ Fe-TA_6_	85.4	label-free, universal, low-cost, and easy to scale-up exosome capture	difficulty in exosome elution	lung-cancer diagnosis and typing	[29]

Ni等^[30]^报道了一种基于双金属-有机框架(MNP/Cu-BTC MOFs)的电化学生物传感器,基于胆固醇标记的DNA链(Chol-DNA)的疏水相互作用识别外泌体。MNP/Cu-BTC MOFs可直接捕获和检测复杂生物样本中的肿瘤来源外泌体,无需使用昂贵的抗体或耗时的离心过程,即可实现对外泌体的超灵敏检测。该生物传感器可一站式实现外泌体的采集、准确鉴定和高灵敏检测,有望加速外泌体分析在常规临床检测中的转化。

### 1.4 基于其他亲和作用的外泌体分离方法

Zhao等^[31]^开发了基于磷脂极性位点印迹策略的磁性磷脂类-分子印迹聚合物(PS-MIPs),基于磁性PS-MIPs技术从尿液中分离和富集所有外泌体,进一步采用两步序贯检测方法构建了双向脂类-分子印迹聚合物-纳米酶联免疫吸附-表面增强拉曼(PS-MIPs-NELISA-SERS)快速超灵敏外泌体检测平台。该平台可实现肿瘤的无创、高灵敏度和快速检测,为肿瘤早期筛查开辟了新途径(图5b)。Wang等^[32]^开发了一种简便、特异性、临床可行的分离-检测一体化生物传感器,用于复杂生物样本中外泌体的分离分析,首次基于硼酸导向的偶联免疫亲和策略实现了肿瘤外泌体的分离-检测。结果表明,该免疫检测平台可以检测痕量样本中的外泌体。此外,该平台还能够破译外泌体的异质性,通过分析外泌体蛋白标志物即可区分4种不同的细胞系。这一策略为无创生物标志物筛查提供了一个强大的工具,具有巨大的临床前景(图5c)。Potrich等^[33]^以氧化硅作为底衬,发展了多种功能化表面。结果表明,负电荷修饰的功能化表面对外泌体的捕获效率最高,可达4×10^8^个/cm^2^。此外,捕获的外泌体可进一步回收并应用于生物标志物检测,如外泌体的microRNA等分析,在基于外泌体的临床诊断和科学研究中具有广泛的应用潜力。

### 1.5 基于微流控技术的亲和分离方法

微流控技术的快速发展为基于微流控设备的开发提供了强大的支持,它可以将样品处理、分析、检测等过程集成于芯片中,具有小型化、集成化、高通量和低时间消耗等优点^[34,35]^。目前,微流控芯片已逐渐成为便捷、高效的外泌体分离工具。

Zheng等^[36]^开发了一种用于特异性外泌体分离的微流控芯片,通过一种新型的PTCDI-适配体信号开关策略集成了敏感的定量分析,交替水滴形微阵列设计提高了外泌体捕获效率(图6a)。该微流控分析平台对HepG2外泌体的检出限低至8.69×10^3^个/mL,线性范围可达6个数量级,为外泌体分析和早期癌症筛查提供了便捷、快速、灵敏的高效平台。Shen等^[37]^设计了一种唾液外泌体两亲性-树突状超分子探针(SEASP)阵列,该阵列能够高效地富集和原位检测外泌体蛋白生物标志物(图6b)。与UC法相比,该方法可获得生理状态和结构良好的高纯度外泌体。结合基于质谱的蛋白质组学分析,筛选出了作为哮喘生物标志物的差异表达蛋白,为液体活检领域的早期疾病筛查和诊断提供了一种快速、低成本、特异性高的筛查流程和实验依据。Liu等^[38]^建立了抗体功能化的脂质膜微阵列,用于识别和捕获癌症特异性外泌体。结果表明,该平台能够在2 h内从未纯化的30~50 μL体液样本中特异性检测和捕获癌症相关的外泌体(图6c)。Han等^[39]^开发了一种尺寸依赖型微流控芯片,通过切向流过滤从人血液中分离纯化外泌体,通过反向洗脱可高效回收微流控芯片中捕获的外泌体(>80%)(图6d)。进一步将收集得到的外泌体进行质谱分析,基于外泌体的指纹峰,验证了外泌体的纯度。与金标准UC法相比,基于尺寸依赖型的微流控芯片技术富集得到的外泌体回收率高,且杂蛋白污染更少。

**图6:**
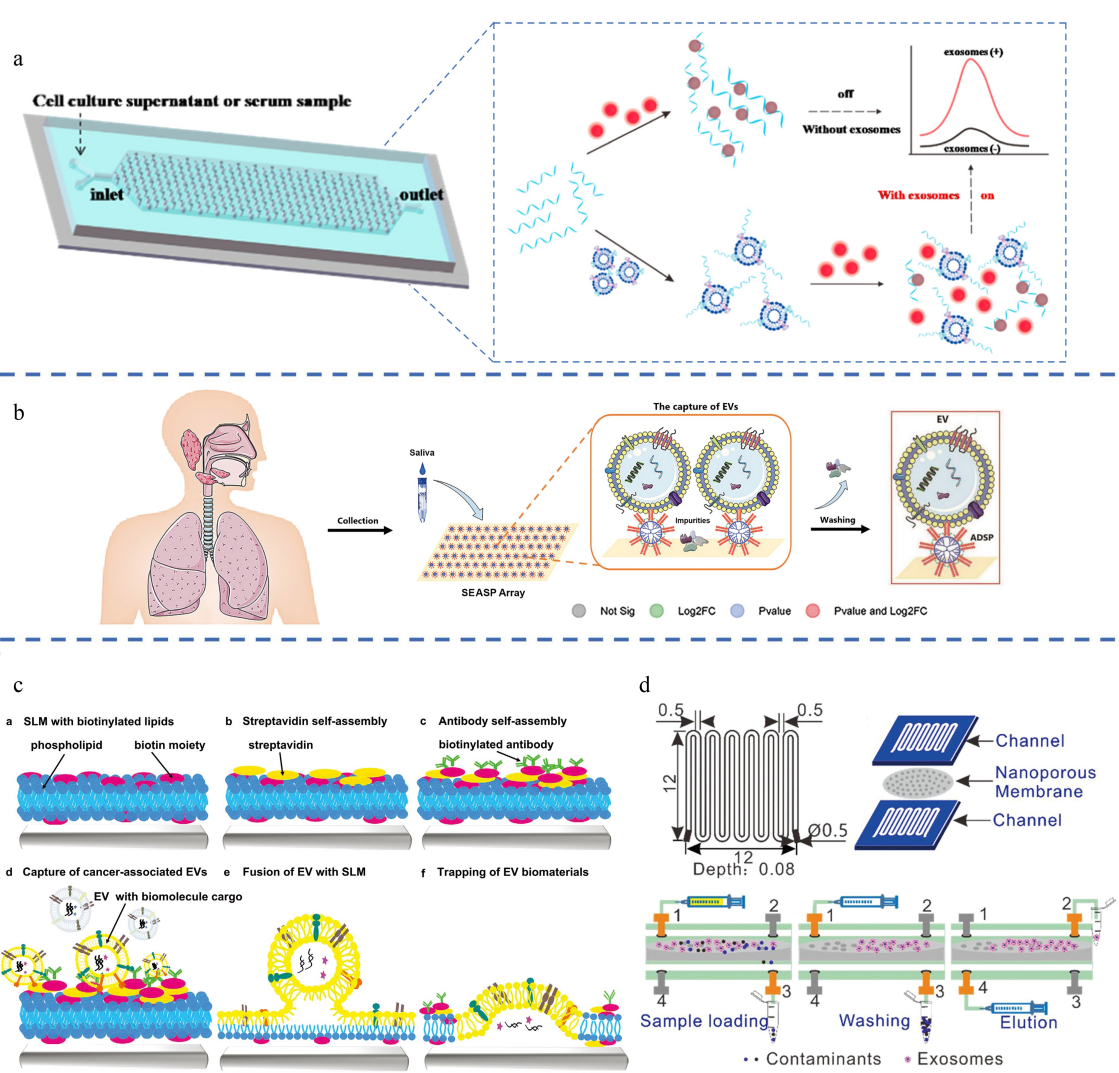
(a)基于微流控芯片的外泌体分过程和苝四羧酸二亚胺衍生物(PTCDI)-适配体荧光信号转导策略用于外泌体检测^[36]^;(b)唾液外泌体蛋白质组学分析和哮喘生物标志物的高通量阵列检测示意图^[37]^; (c)支持脂质膜(SLM)捕获外泌体方案^[38]^; (d)蛇形通道法分离纯化外泌体^[39]^

基于微流控技术的富集方法,可以快速、灵敏地实现外泌体的高纯度和高效富集,已作为重要的分析检测平台应用于各种体液分析。但是,由于其成本较高,对于大样本量的外泌体分离富集,该方法不是最优的选择。此外,苛刻的富集条件也是限制其临床应用的关键因素之一。

## 2 结合亲和富集与常规分离法的外泌体富集策略

### 2.1 UC结合亲和法的富集策略

UC是目前最常用的外泌体分离技术,是外泌体分离富集的金标准。它具有技术成熟度高、样品适用范围广、操作步骤程序化等优点。但该方法分离过程耗时长(>4 h),设备成本高,操作繁杂,对操作人员的经验依赖性高,不同实验室之间的结果重复性差。此外,强大的离心力也会导致外泌体损伤,降低其完整性和生物活性,限制了其在下游研究中的应用。为此,Xiang等^[40]^将UC法与TiO_2_亲和法结合,在20 min内即可实现尿液中低丰度外泌体的高效捕获,其中91.5%以上的外泌体保持了完整的生物结构,鉴定到的蛋白质数量比常规的外泌体富集法提高了23%以上。

### 2.2 超滤法结合亲和法的富集策略

超滤法通过不同孔径的过滤膜,去除杂质从而获得外泌体。该方法操作简单,无需昂贵的特殊设备或添加其他化学试剂,适用于大批量样品中外泌体的分离。然而,该方法中外泌体的回收率常常因滤膜表面的堵塞而降低。为此,Zhang等^[11]^开发了一种三维纸基的外泌体捕获装置(sEV-IsoPD),该装置由用于尺寸过滤的多孔膜和用于免疫亲和力捕获的MOF/抗体修饰的纸芯片组成。sEV-IsoPD结合蠕动泵驱动的流动系统,可从细胞培养液和血清中高效分离外泌体。与超速离心法相比,sEV-IsoPD的产率提高至5.1倍,纯度提高至1.6倍,回收率(77.3%)提高至7.5倍,而所耗时间(30 min)仅为UC法的8.3%,仪器成本(710美元)仅为UC法的1.0%。sEV-IsoPD为外泌体的快速分离和可视化检测提供了一种高效实用的方法,在外泌体的制备和疾病诊断方面具有广阔的应用前景。

### 2.3 尺寸排阻色谱法结合亲和法的富集策略

尺寸排阻色谱法是一种基于样品粒径大小差异的分离技术。该方法简单、高效、经济,适用于大样本处理。Zheng等^[41]^首先采用二维尺寸排阻液相色谱法,根据外泌体粒径差异,初步分离得到了大、中、小外泌体,然后利用开发的亲水性羰基功能化磁性锆有机骨架(CFMZOF)作为探针,特异性捕获翻译后修饰蛋白(PTM)阳性的外泌体。最后,采用液相色谱-串联质谱(LC-MS/MS)结合数据库检索对PTM蛋白含量进行表征,揭示了粒径大小不同的尿液来源外泌体的异质性生物学功能及其作为生物标志物的临床诊断价值。

### 2.4 聚合物沉淀法结合亲和法的富集策略

聚合物沉淀法常被用于分离病毒或其他生物大分子。近年来,该方法已成为外泌体分离的热门方法。其原理是通过向体液样本中添加聚合物从而改变样本中外泌体的分散性,使其沉淀从而达到分离富集的目的。但是,通过聚合物沉淀法富集得到的外泌体容易受到其他生物大分子(脂蛋白或病毒颗粒等)的污染,并可能会干扰后续(蛋白质组学、质谱等)分析。为解决该问题,本课题组^[24]^将聚合物沉淀策略与免疫亲和策略相结合,开发了pH-HGN纳米系统,实现了生理pH条件下外泌体的高效捕获,对于H1299来源的外泌体捕获效率达到90%以上。

## 3 结论

外泌体在肿瘤早期诊断、治疗和预后判断中逐渐显示出巨大应用潜力,已成为液体活检中最有前途的生物标志物。本文综述了基于亲和作用力的外泌体分离富集策略的最新研究进展。其中,免疫亲和法由于特异性强、分离得到的外泌体纯度高,可分离出特异性的外泌体亚类。然而,这种方法大多依赖于能够识别外泌体外表面生物分子的捕获剂,如抗体和适配体等。分离得到的外泌体的质量和纯度受到表面生物活性分子(抗原等)缺失、失活和降解的影响,此外,还存在分离成本高、对试剂和储存条件要求高等缺点,不适合外泌体的大规模捕获应用,可用于特异性外泌体的捕获。基于外泌体膜结构的分离方法以及基于亲疏水作用的富集方法易受到复杂生物体杂质的污染,但富集成本较低、富集过程简单且可避免外泌体本身的异质性造成的低产量,适用于规模化快速分离制备外泌体。一些新兴的分离技术,比如,微流控技术的富集方法,可以快速、灵敏地实现外泌体的高质量富集,但成本较高。此外,苛刻的富集条件也是限制其临床应用的关键因素之一。随着新的靶点结构和活性分子的发现,新的配体比现有的配体具有更高的选择性或更好的解离常数成为外泌体亲和分离法关注的焦点。综上,由于外泌体具有低浓度和基质复杂等特点,尚未有标准的临床外泌体分离富集方法,未来仍有大量的研究工作值得探索。

## 作者团队简介

药物分析与医药功能材料团队隶属于河北大学药学院,在乔晓强教授的带领下,自2011年以来,课题组致力于新型分离富集材料、高灵敏检测技术开发及在生物医药领域中的应用基础研究工作,承担了多项国家级科研项目,并与相关领域学者建立了良好的合作关系。

**Table:** 

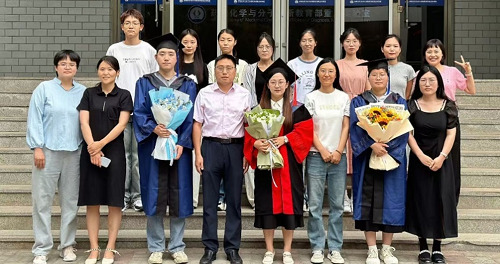	人才队伍
	**课题组组长:**乔晓强教授 **职工及学生:**教授1人,副研究员1人,讲师3人,硕博研究生20余人 **团队精神:**科研载道,砥砺深耕

**Table:** 

科研项目及成果	研究领域
**科研项目:**国家自然科学基金,河北省自然科学基金,中国博士后科学基金,河北省科技支撑计划,河北大学创新团队计划 **科研成果:**在*Nature Biomedical Engineering*、*Analytical Chemistry*、*ACS Applied Materials & Interfaces*、*TrAC-Trends in Analytical Chemistry*、*Journal of Chromatography A*等学术期刊发表SCI论文60余篇,授权发明专利6项,在科学出版社出版和化学工业出版社出版《药学文献检索》《一学就会的中医学基础》《药学文献检索与利用》等3部教材 **获奖情况:**省部级科技进步二等奖